# Establishment and Application of Multiple Cross Displacement Amplification Coupled With Lateral Flow Biosensor (MCDA-LFB) for Visual and Rapid Detection of *Candida albicans* in Clinical Samples

**DOI:** 10.3389/fcimb.2019.00102

**Published:** 2019-04-16

**Authors:** Fan Zhao, Lina Niu, Linlin Yan, Jinqing Nong, Chunmei Wang, Jing Wang, Naishu Gao, Xiaoxue Zhu, Lei Wu, Fengzhi Zheng, Shoukui Hu

**Affiliations:** ^1^Department of Clinical Laboratory, Peking University Shougang Hospital, Beijing, China; ^2^Department of Pathogen Biology, School of Basic Medicine and Lifescience, Hainan Medical University, Haikou, China; ^3^Key Laboratory of Translation Medicine Tropical Diseases, Haikou, China; ^4^Hainan Medical University-University of Hong Kong Joint Laboratory of Tropical Infectious Diseases, Haikou, China

**Keywords:** *Candida albicans*, opportunistic pathogenic yeast, multiple cross displacement amplification, gold nanoparticle-based lateral flow biosensor, loop-mediated isothermal amplification, qPCR, clinical samples

## Abstract

*Candida albicans* is an opportunistic pathogenic yeast that predominantly causes invasive candidiasis. The conventional diagnosis of *C. albicans* infection depends on time-consuming, culture-based gold-standard methods. Here, a multiple cross displacement amplification (MCDA) assay, combined with a gold nanoparticle-based lateral flow biosensor (LFB) visualization method, was developed for the rapid detection of *C. albicans*. The internal transcribed spacer II, a region between 5.8 and 28 S fungal ribosomal DNA, is a *C. albicans* species-specific sequence that was used as the MCDA assay target. As an isothermal amplification method, the MCDA reaction with optimized conditions could be completed within only 40 min at a constant temperature (64°C). Then, the amplification reaction products could be visibly detected by a LFB without special equipment. The developed MCDA-LFB assay for *C. albicans* detection was a specific and accurate method, and could distinguish *C. albicans* from other pathogens. Just 200 fg of genomic DNA template from pure cultures of *C. albicans* could be detected using the MCDA-LFB method. The limit of detection (LOD) of the new method was more sensitive than that of both qPCR and loop-mediated isothermal amplification (LAMP). Of 240 clinical sputum samples, all of the *C. albicans*-positive (87/240) samples identified by the gold-standard method were successfully detected by the MCDA-LFB assay. Moreover, the true positive rate of the newly developed assay was not only higher than that of qPCR (100 vs. 86.2%), but also higher than that of LAMP (100 vs. 94.3%). Thus, the MCDA-LFB assay might be a simple, specific, and sensitive method for the rapid diagnosis of *C. albicans* in clinical samples.

## Introduction

*Candida albicans* is a member of the human gut flora and it is also an opportunistic pathogenic yeast (Gow and Yadav, 2017). *C. albicans*, together with other species of Candida such as *C. tropicalis, C. parapsilosis*, and *C. glabrata*, are responsible for 50–90% of all cases of candidiasis in humans (Pfaller and Diekema, 2007; Martins et al., 2014; Schlecht et al., 2015). As one of the most common agents responsible for invasive candidiasis, *C. albicans* infection has been reported to result in a mortality rate of 40% for patients with systemic candidiasis. From an estimated report, invasive candidiasis contracted in a hospital setting could cause up to 11,200 deaths annually in the United States (Pfaller and Diekema, 2007).

However, the current gold-standard method for *C. albicans* detection is based on phenotyping, including culture, microscopic examination, and biochemical identification. It typically requires more than 2 days for growth and identification, which is limited by slow turn-around times; therefore, positive identification may occur late in the course of infection. Such delayed diagnosis may cause poor outcomes in patients with systemic fungal disease. Thus, it is imperative to develop and validate a rapid and accurate method for *C. albicans* identification. Several non-culture methods have been developed for *C. albicans* detection, including mass spectrometry (Zehm et al., 2012), immunoassay (Gunasekera et al., 2015), polymerase chain reaction (PCR) (Vahidnia et al., 2015), real-time PCR (Maaroufi et al., 2003; Kasai et al., 2006), polymerase spiral reaction (PSR) (Jiang et al., 2016), and loop-mediated isothermal amplification (LAMP) (Noguchi et al., 2017). Compared with the gold-standard culture method, these tests for *C. albicans* identification can save considerable time. However, such assays conversely depend on technical expertise or complex equipment, which may not be available in some settings.

Multiple cross displacement amplification (MCDA) is an isothermal amplification technology similar to LAMP, but with more sensitivity in bacterial detection (Wang et al., 2015). MCDA assays have been applied to detect several species of bacteria including *Vibrio parahaemolyticus* (Wang et al., 2016a), *Shigella* spp.(Wang et al., 2016b), *Listeria monocytogenes* (Wang et al., 2015), and *Pseudomonas aeruginosa* (Zhao et al., 2018). On the other hand, detection of amplification products from MCDA can be performed by gold nanoparticle-based lateral flow biosensors (LFBs). The LFB is a novel method to detect specific DNA fragments, which is based on the binding of antibodies (embedded on the LFB) and haptens (labeled on the 5′ side of primers) (Ang et al., 2015) (Chen et al., 2016; Toubanaki et al., 2016). In comparison with other methods, such as gel electrophoresis, with colorimetric indicators, and real-time turbidity for indicating MCDA results, LFB is relatively quick, objective, and simple (Wang et al., 2016a,b; Zhao et al., 2018). Therefore, a combined MCDA and LFB (MCDA-LFB) method could be used as an easy, rapid, specific, and very sensitive test for microbial detection. Internal transcribed spacer II (ITS II), a region between 5.8 and 28 S fungal ribosomal DNA, is frequently used for identification of fungal species, as the ITS II region of fungi exhibits sequence variability (Turenne et al., 1999; Jiang et al., 2016). Here, we aimed to establish a MCDA-LFB assay for *C. albicans* detection via the species-specific sequence from ITS II region with DNA template from pure cultures of the reference strain, following it, we applied the assay to clinical samples. We also compared the developed MCDA-LFB method with the gold-standard method and the more-commonly used molecular methods, in order to explore the application of our method in clinical practice.

## Materials and Methods

### Fungi, Bacteria Strains, and Clinical Samples

The 37 fungi strains and 2 bacteria strains used in this study are listed in Table 1, including 13 *C. albicans* and 26 non-*C. albicans* strains. Two reference strains were from American Type Culture Collection (ATCC), including one *C. albicans* strain (ATCC14053) and one *Klebsiella pneumoniae* strain (ATCC2146). Other strains were isolated from clinical samples, which were identified by the gold-standard method in the Clinical Laboratory of Peking University Shougang Hospital. All strains were stored in 15% (w/v) glycerol broth at –70°C after pure culture isolation. DNA from *C. albicans* ATCC14053 was used as a positive control, mainly for analysis of assay performance, and for determination of the optimal reaction temperature, while DNA from *K. pneumoniae* ATCC2146 and *Pseudomonas aeruginosa* SGH-PA001 strains were used as negative controls. To assess the limit of detection (LOD), DNA of *C. albicans* ATCC14053 was serially diluted, as indicated in the results section. DNA from 13 *C. albicans* strains and 26 non-*C. albicans* strains were used to analyze the specificity of the developed method.

**Table 1:** Fungi and bacteria strains used in this study.

**ID**	**Strains**	**Strain No.^a^**
1	*Candida albicans*	ATCC14053
2–13	*Candida albicans*	SGH-CA001~012
14–18	*Candida tropicalis*	Isolated strains (SGH)
19–22	*Candida parapsilosis*	Isolated strains (SGH)
23–27	*Candida glabrata*	Isolated strains (SGH)
28–30	*Candida krusei*	Isolated strains (SGH)
31	*Candida dubiniensis*	Isolated strains (SGH)
32	*Candida rugosa*	Isolated strains (SGH)
33	*Candida guilliermondii*	Isolated strains (SGH)
34	*Cryptococcus neoformans*	Isolated strains (SGH)
35	*Cryptococcus gattii*	Isolated strains (SGH)
36	*Cryptococcus luteolus*	Isolated strains (SGH)
37	*Cryptococcus curvatus*	Isolated strains (SGH)
38	*Klebsiella pneumoniae*	ATCC2146
39	*Pseudomonas aeruginosa*	SGH-PA001

a *SGH, Shougang Hospital; ATCC, American Type Culture Collection; CA, Candida albicans; PA, Pseudomonas aeruginosa*.

To explore the applicability of the *C. albicans* MCDA-LFB assay to clinical samples, human sputum samples were serially collected from January 1st to August 31st 2018 in the Clinical Laboratory, Peking University Shougang Hospital. This study was approved by the Ethics Committee of Shougang Hospital, and conducted according to the regulations of the Ministry of Health, China. Two hundred and forty human sputum samples were collected and 87 from the 240 samples were identified as *C. albicans*-positive by the gold-standard methods. All collected samples were stored at –70°C until use. The protocol was approved by the Ethics Committee of Shougang Hospital. Patients who provided sputum samples gave written informed consent, in accordance with the Declaration of Helsinki.

### Genomic DNA Extraction

*C. albicans* strains and non-*C. albicans* fungi strains were subcultured on Emmons' modification of Sabouraud's agar plates at 30°C for 48 h before DNA extraction, while bacteria strains were subcultured on nutrient agar plates at 37°C. Genomic DNA was extracted from each strain using a DNA extraction kit (QIAamp DNA Mini Kits, Qiagen, Beijing, China) in accordance with the manufacturer's instructions. DNA concentration was measured by spectrophotometry using a NanoDrop 1,000 (Thermo Fisher Scientific, Wilmington, DE, USA). For clinical samples, DNA was extracted using the same QIAamp DNA extraction kit after the stored samples were thawed, in accordance with the manufacturer's instructions. The extracted DNAs were stored at −20°C until use.

### The MCDA-LFB Assay for *C. albicans* Detection

**Figure 1:**
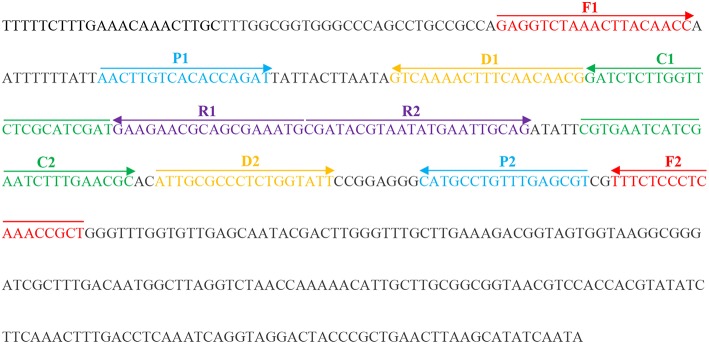
Appropriate primer design for *C. albicans* MCDA-LFB assay and primer sequence positions in the selected fragment including *ITS II*.

According to the standard MCDA method (Wang et al., 2015), a set of 10 primers encompasses 10 distinct regions from one selected fragment, including the species-specific sequence. In this study, we designed three sets of MCDA primers targeting three fragments of the selected species-specific sequence of *C. albicans* (Table S1). All primers were designed using PrimerExplorer V4 (Eiken Chemical) and primer software PRIMER PREMIER 5.0. The locations and sequences of the MCDA primers are shown in Figure 1 and Table 2.

**Table 2:** Primers used for MCDA, LAMP and qPCR assays.

**Primers name**	**Sequences and modifications (5^′^-3^′^)**	**Length (bp)**
**MCDA ASSAY PRIMERS**		
F1	GAGGTCTAAACTTACAACC	19
F2	AGCGGTTTGAGGGAGAAA	18
CP1	ATCGATGCGAGAACCAAGAGAT CAACTTGTCACACCAGAT	40
CP2	CGTGAATCATCGAATCTTTGAAC GCACGCTCAAACAGGCATG	42
C1	ATCGATGCGAGAACCAAGAGATC	23
C1-FITC	5′-FITC-ATCGATGCGAGAAC CAAGAGATC-3′	23
C2	CGTGAATCATCGAATCTTTGAACGC	25
D1	CGTTGTTGAAAGTTTTGAC	19
D1-Biotin	5′-Biotin-CGTTGTTGA AAGTTTTGAC-3 ′	19
D2	ATTGCGCCCTCTGGTATT	18
R1	CATTTCGCTGCGTTCTTC	18
R2	CGATACGTAATATGAATTGCAG	22
**LAMP ASSAY PRIMERS (PATENT NO. WO 2014/133153 A1)**		
Calb-F3	TCTGGTATTCCGGAGGGC	18
Calb-B3	AGTCCTACCTGATTTGAGGT	20
Calb-FIP	CTACCGTCTTTCAAGCAAACCC ATGAGCGTCGTTTCTCCCT	41
Calb-BIP	TTGACAATGGCTTAGGTCTAACC AAAAGATATACGTGGTGGACGTTAC	48
Calb-LF	CTCAACACCAAACCCAGCGG	20
**qPCR PRIMERS (Alonso et al., 2018)**		
CA-qPCR-F	ATTCGGTGAGTAATCCTA	18
CA-qPCR-R	GTATAGTCCAGATAACAACA	20

The standard MCDA reaction mixture was 25 μl with the compound as described previously (Wang et al., 2016a,b) with some modification (Zhao et al., 2018). In brief, the reaction mixture included: 2 × reaction mix (Isothermal Amplification Kit, Jieyite, Tianjin, China), 12.5 μl; displacement primers F1 and F2, 0.4 μM each; amplification primers C1 and C2, 0.8 μM each; amplification primers R1, R2, D1, and D2, 1.2 μM each; cross primer CP1,1.2 μM; cross primer CP2, 2.4 μM; *Bst* DNA polymerase, 1.25 μl (10 U); and DNA template, 1 μl. Negative control mixtures contained 10 ng genomic DNA of *K. pneumoniae* ATCC2146 or *P. aeruginosa* SGH-PA001, and blank controls used 1 μl double distilled water. For LFB detection, primer C1, labeled with fluorescein isothiocyanate (FITC), and biotin-labeled primer D1, were used in the MCDA reaction instead of unlabeled ones. All of the primers were produced and purified by Ruiboxingke Biological Technology Ltd (Beijing, China).

Amplicons were analyzed by one or more of four different methods: (1). 2% agarose gel electrophoresis; (2). a colorimetric indicator (malachite green, MG); (3). by using a turbidimeter (Loopamp Realtime Turbidimeter LA-320C, Tokyo, Japan); (4), or by LFB. We constructed a gold nanoparticle-based dipstick biosensor, according to the description in a previous paper (Wang et al., 2016b) (Wang et al., 2016a). For electrophoresis, reaction products were separated by applying a voltage of 5 volts/cm across the gel for at least 30 min. DNA amplification products were visualized by exposing the gel to UV light; a specific ladder of bands is observed for positive amplifications, but not for negative controls. When using MG, the amplified products cause a change in the solution color, from colorless to sky blue (negative/blank controls remain colorless). With LFB, two red lines, including the test line (the lower one), and the control line (the upper one), are observed for positive reactions, but the control line only is visualized for negative and blank control reactions.

The process for assessing and verifying the effectiveness of the primers we designed was as described previously (Zhao et al., 2018). Briefly, the reactions were initially set at 62°C for 40 min and then amplification was terminated by heating at 85°C for 5 min. The optimal reaction temperature for *C. albicans*-MCDA assay was then determined in reactions with the most effectiveness, using one of the three sets primers at fixed temperatures starting from 60 to 65°C with intervals.

### The Specificity and Sensitivity of MCDA-LFB Assay for *C. albicans* Detection

The specificity of the developed assay for *C. albicans* detection was analyzed using DNA from 39 strains, including 13 *C. albicans* strains, 24 non-*C. albicans* fungi strains, and 2 bacteria strains (Table 1). Except *C. albicans*, other microbe including the fungi and bacteria species used in this study are also common in hospitals. The sensitivity of the MCDA-LFB assay indicates the detection limit. This was defined in terms of the amount of genomic DNA template. Genomic DNA template from ATCC14053 was serially diluted from 2 ng/μl to 2 fg/μl (2 ng/μl, 20 pg/μl, 2 pg/μl, 500 fg/μl, 200 fg/μl, 20 fg/μl, and 2 fg/μl). The detection of *C. albicans-*MCDA by LFB was compared to those using the other three methods (see above), with three replicates.

To compare the sensitivity of MCDA with that of LAMP and qPCR assays for pure cultures, template DNA from the reference strains was diluted from 2 ng/μl to 2 fg/μl. For LAMP assays, the Loopamp Specified Microorganism “Fungi Candida albicans” Detection Kit (Eiken Chemical Co., Tokyo, Japan) was used in accordance with the manufacturer's instructions (Noguchi et al., 2017). In brief, the primer sequence for LAMP assay is targeting ITS region, which has obtained the patent NO. WO 2014/133153 A1 (Table 2). DNA amplification and real-time monitoring of the sample turbidity were conducted using the turbidimeter (LA-320C), for 40 min at 65°C. For qPCR assays, primers and amplification procedures were as described previously (Alonso et al., 2018). The primer sequence is targeting ACT1 gene (Table 2). The cycling protocol were: 5 min at 95°C, 39 cycles of denaturation during 15 s at 94°C, annealing at 55°C for 30 s, and elongation at 60°C for 15 s, with melt curve tested with gradual increase of 0.5°C from 55 to 95°C.

### Application of *C. albicans-*MCDA-LFB Technology for Clinical Samples

Two Hundred and Forty human clinical sputum specimen were used to assess the applicability of our *C. albicans*-MCDA-LFB technology for clinical samples. By the gold-standard method, 87 (36.3%) of the 240 samples were identified as *C. albicans*-positive, while the remaining 153 samples were identified as *C. albicans*-negative, including samples with other fungi or bacteria growing, or microbe-free samples. The true positive rate, true negative rate, and consistency rate of results from *C. albicans*-MCDA-LFB, qPCR, or LAMP assays were computed based on the result from gold-standard method.

### Statistical Method

True positive rate of the MCDA-LFB, qPCR, or LAMP assays for clinical sample detection was computed by determining the number of true positive/(number of true positive + number of false negative) × 100%. True negative rate of the MCDA-LFB, qPCR, or LAMP assays for clinical detection was computed by determining the number of true negative/(number of true negative + number of false positive) × 100%. Consistency rate of the MCDA-LFB, qPCR, or LAMP assays for clinical sample detection was computed by determining the (number of true positive + number of true negative)/number of all samples × 100%.

## Results

### Detection of *C. albicans*-MCDA Products

**Figure 2:**
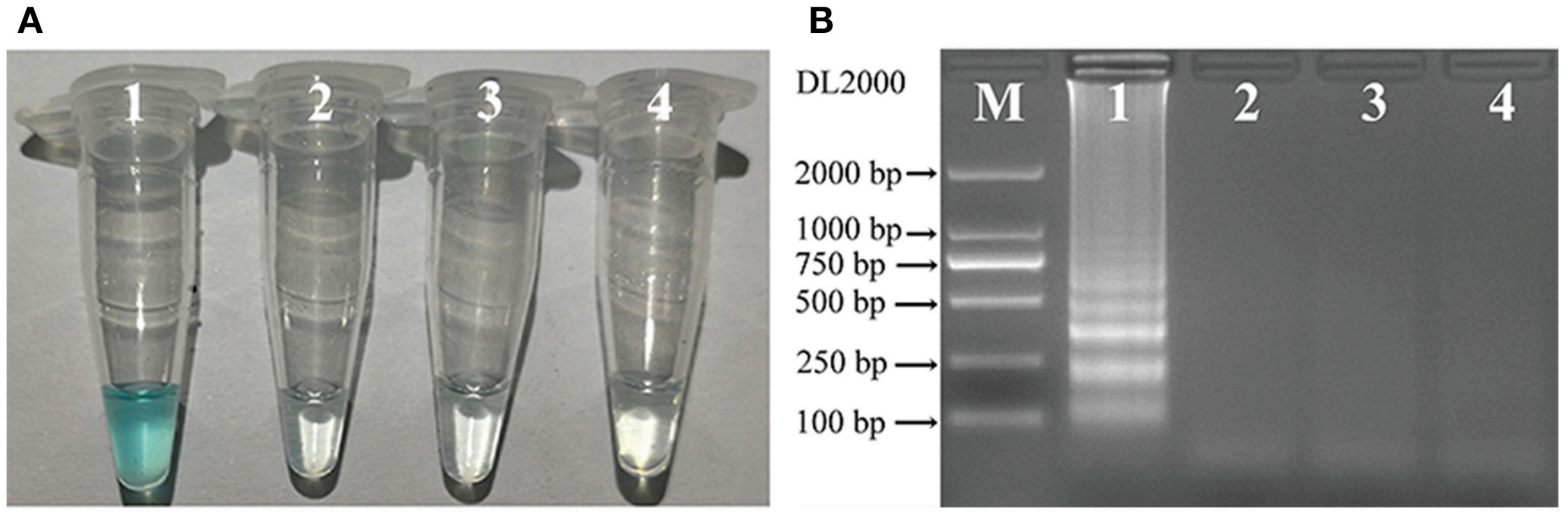
Detection of *C. albicans*-MCDA products with the appropriate primers. **(A)** Using MG, amplification products in the *C. albicans*-MCDA assay were detected visually by a color change (sky blue for positive samples, colorless for negative samples); **(B)** 2% agarose gel electrophoresis of *C. albicans*-MCDA products. Lane M, DNA maker DL2000. Tube 1/Lane 1: amplification of *C. albicans* strain ATCC14053; Tube 2/Lane 2: amplification of *Klebsiella pneumoniae* strain ATCC2146 (negative control); Tube 3/Lane 3: amplification of *Pseudomonas aeruginosa* SGH-PA001 (negative control); Tube 4/Lane 4: double distilled water (blank control). MCDA, multiple cross displacement amplification; MG, Malachite Green; LFB, lateral flow biosensor.

To identify the most effective of the three sets of primers for *C. albicans*, MCDA assays were performed at 62° for 40 min using DNA from pure cultures as template. The most effective set of primers was selected by inspection of the curves from real-time turbidity (LC320) for use as future experimental primers (data not shown). Then, to confirm the effectiveness of the MCDA primers we selected (Table 1; Figure 1), the amplification products were detected by two different methods, as mentioned in the Methods section. Of the two methods, results were observed to be positive when the template was DNA from *C. albicans* ATCC14053, but not with DNA from *K. pneumoniae* ATCC2146 or *P. aeruginosa* SGH-PA001 (negative controls), or from the blank control (Figure 2). Therefore, the *C. albicans*-MCDA primer set we selected in the current study was suitable for MCDA-LFB assay development.

### Optimal Assay Temperature

**Figure 3:**
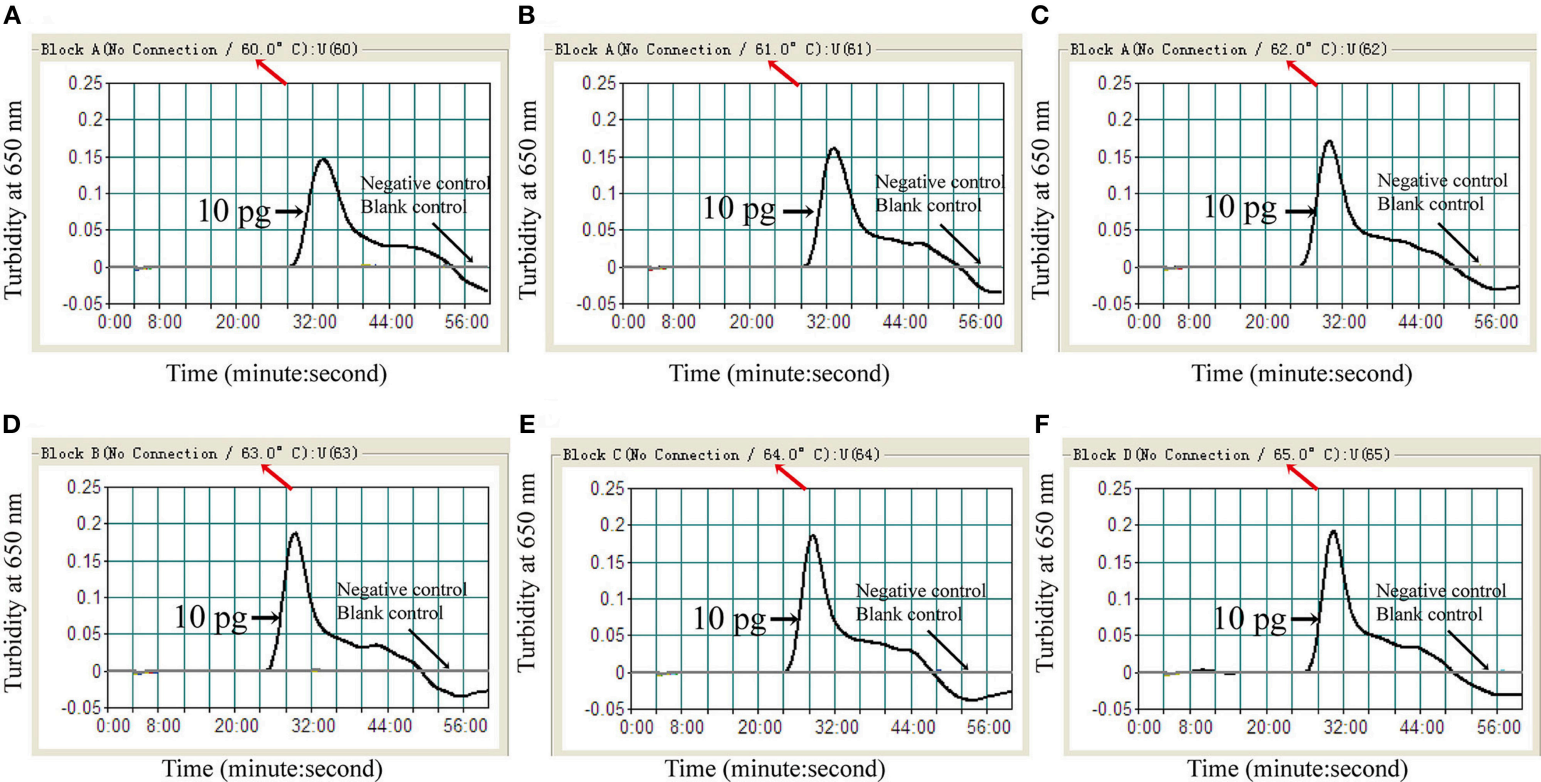
Optimal amplification temperature for *C. albicans*-MCDA assay. *C. albicans*-MCDA reactions were monitored by real-time measurement of turbidity. The corresponding curves are shown in the panels. *K. pneumoniae* and sterile double-distilled water were used as a negative control, and a blank control, respectively. Turbidity of >0.1 was considered a positive reaction. Six kinetic curves **(A–F)** were generated from 60 to 65°C, with 10 pg *C. albicans* DNA per reaction. MCDA, multiple cross displacement amplification.

To identify the optimal assay temperature, the MCDA assay was conducted from 60 to 65°C with 10 pg/reaction of *C. albicans* ATCC14053 DNA as template. All reactions were monitored by real-time turbidity (LC320). The kinetic data in Figure 3 show that 64°C was the optimum reaction temperature for the selected primers for detection of *C. albicans* by MCDA.

### Specificity of the *C. albicans*-MCDA-LFB

**Figure 4:**
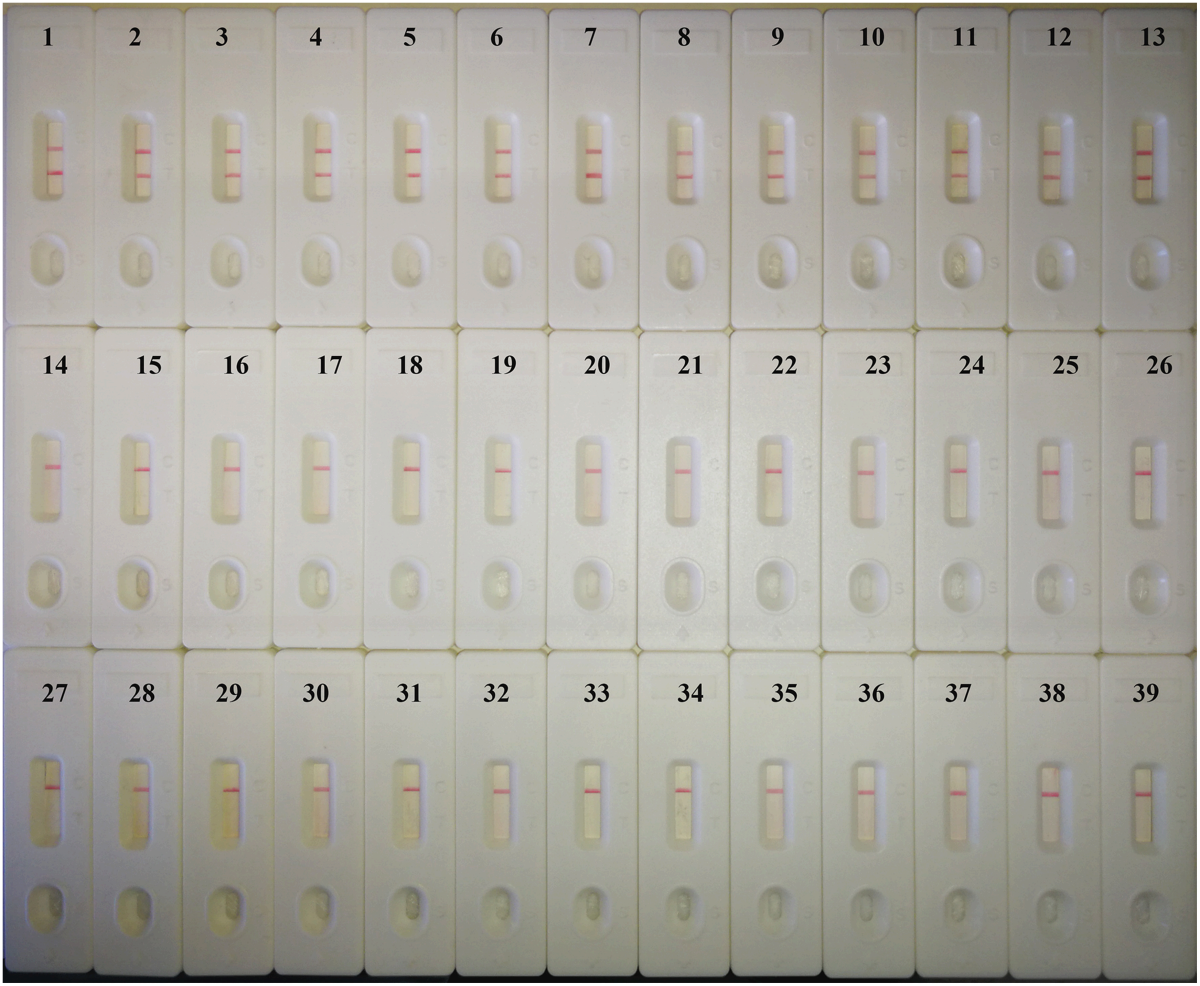
Specificity of the MCDA-LFB detection assay for different strains. Biosensor 1, *C. albicans* ATCC14053; Biosensor 2–13, different *C. albicans* strains isolated from clinical samples; Biosensor 14–18, *Candida tropicalis*; Biosensor 19–22, *Candida parapsilosis*; Biosensor 23–27, *Candida glabrata*; Biosensor 28–30, *Candida krusei*; Biosensor 31–39, *Candida dubiniensis, Candida rugosa, Candida guilliermondii, Cryptococcus neoformans, Cryptococcus gattii, Cryptococcus luteolus, Cryptococcus curvatus, Klebsiella pneumoniae, Pseudomonas aeruginosa*.

When DNA from 37 fungi strains and 2 bacteria strains was tested by MCDA-LFB assay, only 13 *C. albicans* DNA showed positive results. Amplification products were not detected when the template was DNA from non-*C. albicans* strains (Figure 4). Therefore, the MCDA-LFB was highly specific for *C. albicans* detection.

### Sensitivity of the *C. albicans*-MCDA Assay

**Figure 5:**
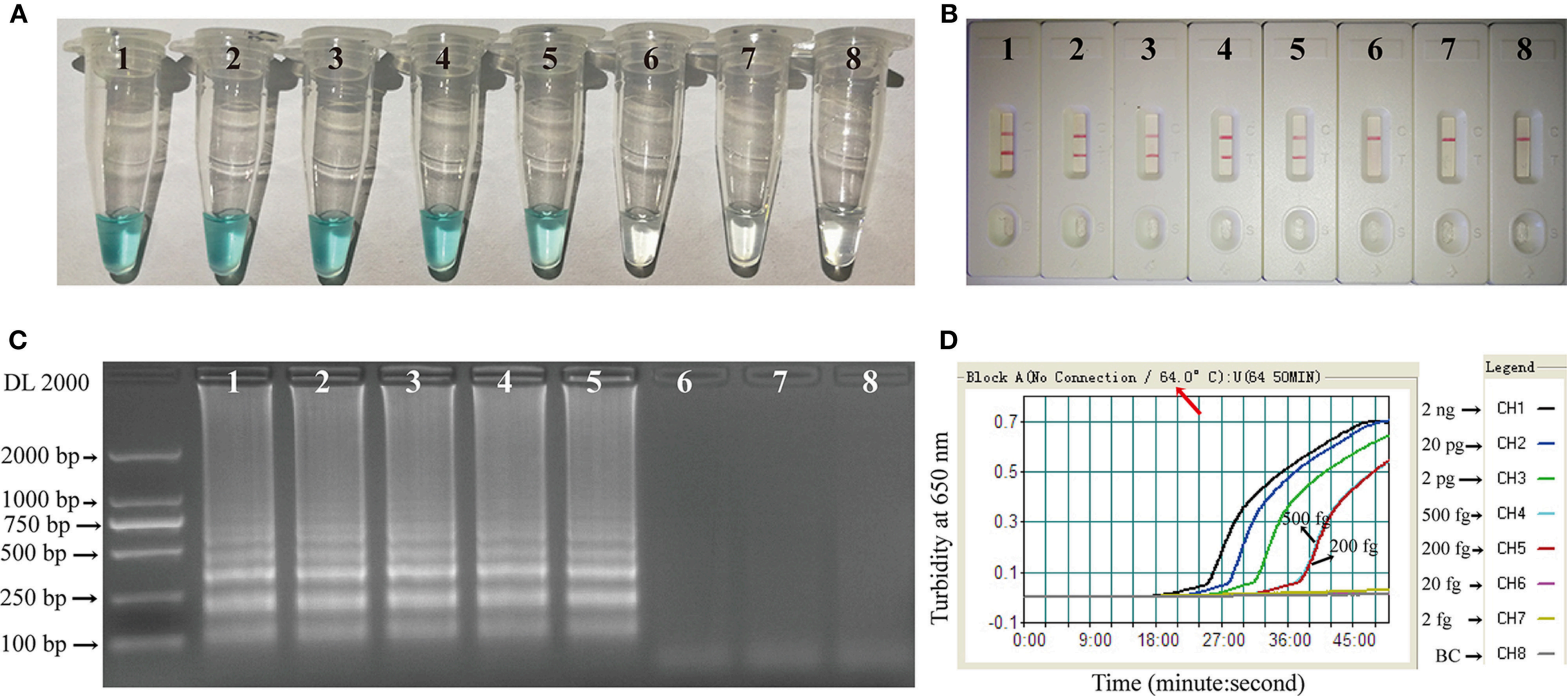
Sensitivity of the *C. albicans*-MCDA assay. Four detection techniques, including a colorimetric indicator (MG) **(A)**, lateral flow biosensor **(B)**, gel electrophoresis **(C)**, and real-time turbidity **(D)**, were applied for analyzing amplification products. Serial dilutions (2 ng, 20 pg, 2 pg, 500 fg, 200 fg, 20 fg, and 2 fg) of target template DNA were subjected to standard MCDA reactions. Tubes **(A)**/Lanes **(B)**/Biosensor **(C)**/Turbidity signals **(D)** 1–7 represent the template DNA mass levels of 2 ng, 20 pg, 2 pg, 500 fg, 200 fg, 20 fg, and 2 fg per reaction, respectively; 8 represents a blank control. MG, malachite green; MCDA, multiple cross displacement amplification.

Two Hundred femtogram of *C. albicans* template DNA yielded amplicons that could be detected colorimetrically, by LFB, by electrophoresis, or by turbidity (Figure 5). The results obtained using the LFB (Figure 5B) agreed completely with those by the other three methods. The MCDA assay was 2.5-fold more sensitive than LAMP for the detection of *C. albicans* by LFB, and was 5-fold more sensitive than qPCR for the detection of *C. albicans* by electrophoresis (Table 3).

**Table 3:** Limit of detection (LOD) and amplification time for detection of *C. albicans* using MCDA-LFB, LAMP, and qPCR.

**Method**	**LOD (DNA/Reaction)**	**Amplification time (min)**
MCDA-LFB	200 fg	40
LAMP	500 fg	40
qPCR	1,000 fg	60

### Evaluation of Clinical Samples for MCDA-Based Identification of *C. albicans*

A total of 240 clinical sputum samples were assayed using *C. albicans-*MCDA-LFB and qPCR (Table 4). By the gold-standard method, 87 of the 240 clinical samples were positive for *C. albicans*. The MCDA-LFB assay results were in complete agreement. However, it was identified that there were 82 of 87 positive samples by LAMP, and 75 of 87 positive samples by qPCR assays. Thus, the true positive rate and true negative rate for *C. albicans-*MCDA-LFB assay were both 100%, while they were 94.3 and 100% for LAMP, 86.2 and 100% for qPCR, respectively.

**Table 4:** Clinical application of *C. albicans*-MCDA-LFB assay in sputum samples.

**Method**	**Result**	**Gold-standard method**		**True positive rate**	**True negative rate**	**Consistency rate**
		**+**	**–**			
MCDA-LFB	**+**	87	0	100.0%	100.0%	100.0%
	**–**	0	153			
LAMP	**+**	82	0	94.3%	100.0%	97.9%
	**–**	5	153			
qPCR	**+**	75	0	86.2%	100.0%	95.0%
	**–**	12	153			

## Discussion

*C. albicans*, the most common human fungal pathogen causing diseases ranging from mucosal to systemic infections, is an important nosocomial, infectious pathogen. Culture based microscopic examination and biochemical identification is the gold-standard method for identification of *C. albicans* infections, but is labor-intensive and minimally effective. Thus, early and rapid identification of *C. albicans* in clinical samples of patients will help to control infection by this pathogen. To date, many techniques other than the culture based method have been developed to detect *C. albicans* in clinical samples (Maaroufi et al., 2003; Kasai et al., 2006; Zehm et al., 2012; Gunasekera et al., 2015; Vahidnia et al., 2015; Jiang et al., 2016; Noguchi et al., 2017). However, to the best of our knowledge, the present study is the first report of a MCDA-LFB assay developed to identify *C. albicans*, with a LOD for *C. albicans* as low as 200 fg of DNA from pure cultures.

Compared to qPCR, the developed MCDA-LFB method is less dependent on equipment and more sensitive. As an isothermal amplification assay, MCDA only requires a simple water bath or heater. Moreover, there were 5 pairs of primers engaged on the target-specific sequence in our MCDA method, which make it more effective than the normal PCR method. In the present study, the PCR method had less true positive rate than MCDA when both were applied for *C. albicans* detection in clinical samples. We also compared MCDA with other isothermal amplification assays, such as LAMP, for *C. albicans* detection. The primers for LAMP were obtained from Noguchi's report (Noguchi et al., 2017). By their method, the LOD to detect *C. albicans* was 1 pg. Here, we reported the LOD was 500 fg for LAMP to detect *C. albicans*, while it was 200 fg for MCDA. These results showed that MCDA was 2.5-fold more sensitive than LAMP in our study, in accordance with previous studies (Wang et al., 2015). On the other hand, the positive reactions could be obtained in as rapidly as 25 min in real-time turbidity measurements. These results indicated that the MCDA is a sensitive and rapid alternative to other molecular techniques for detection of trace amounts of *C. albicans*.

In the present study, the objective, rapid, and simple gold-nanoparticle-based LFB was also introduced to “read” the amplification products. LFB could detect amplification products labeled with FITC and biotin, through the appearance of red lines on the LFB strips within 2 min. All of the *C. albicans*-positive products from MCDA were successfully identified using the LFB, which indicates that the LFBs are relatively more objective and reliable than turbidimetric and colorimetric indicators [9]. On the other hand, although the gel electrophoresis method could distinguish specific amplification from non-specific amplification, it is laborious and requires specific, dedicated equipment. Considering the high workload involved in diagnostic examinations in clinical laboratories, LFB represents a promising candidate to detect the amplification products of either normal PCR or isothermal amplification approaches.

Importantly, we also successfully applied the developed *C. albicans* MCDA-LFB assay to clinical sputum samples. When compared with the time-consuming and labor-intensive gold-standard method, the new assay is a rapid and easy-to-perform method, with 100% true positive rate and 100% true negative rate. In particular, its true positive rate is higher than that of the commonly used qPCR (100 vs. 86.2%). The entire *C. albicans*-MCDA-LFB procedure, including sample processing, could be completed within 2 h. This is much faster than the culture-based method. Our study suggests that the MCDA-LFB assay could be used for fast *C. albicans* detection in clinical sputum samples. In the near future, we will focus on other clinical sample types, e.g., urine, blood, and throat swabs, to test the clinical application of the *C. albicans*-MCDA-LFB assay in diverse samples.

There are also several possible limitations to our study, which should be addressed. First, the developed MCDA-LFB assay is a qualitative determination for *C. albicans*, as we could not quantify the amount of pathogen in the sample, whereas the culture-based method is also a qualitative determination for pathogen detection. Quantification of the amount of microbe in samples may be the direction of pathogen detection research. This is because if it is possible to quantify the amount of microbe in samples, we could monitor and evaluate the therapeutic effect of antibiotics. Second, for isothermal amplification methods with high concentrations of primers, carry-over contamination is a great concern for genomic template-based identification assays. A possibility to lower carryover contamination is to control the amplification time, which has been suggested by Wang et al. (2018). In this study, the amplification time was also limited to 40 min to reduce false-positive results. Moreover, we strictly controlled the laboratory environment to reduce the production of aerosols in experimental processes. It was recently reported that carry-over contamination could be removed by adding Antarctic thermolabile uracil-DNA-glycosylase (UNG) into the reaction system before amplification with the mixture of dTTP and dUTP instead of dTTP alone (Wang et al., 2017, 2018). Although UNG was not added to the reaction system in this study, the detection results were the same as the gold-standard methods in the evaluation of clinical samples for MCDA-based identification of *C. albicans*, indicating that false positive rates have been controlled in our laboratory. Certainly, it would be a good choice to further reduce carry-over contamination through adding Antarctic thermolabile UNG into the reaction system before MCDA assay performance.

In conclusion, we developed a MCDA-LFB assay for rapid and effective detection of *C. albicans*. The LOD of the new assay for *C. albicans* detection from pure cultures was as low as 200 fg. In comparison to other molecular methods (PCR, LAMP) for detection of *C. albicans*, the MCDA-LFB method is more sensitive, time-saving, and does not require expensive equipment or diagnostic technology. When using the MCDA-LFB assay to detect infected clinical samples, the results were consistent with those of the gold-standard method. This suggests the possibility for further clinical application of our *C. albicans*-MCDA-LFB assay.

## Ethics Statement

This study was approved by the Ethics Committee of Shougang Hospital, and conducted according to the regulations of the Ministry of Health, China. The protocol was approved by the Ethics Committee of Shougang Hospital. Patients who provided sputum samples gave written informed consent, in accordance with the Declaration of Helsinki.

## Disclosure

SH, FaZ, LN, LY, JN, FeZ, NG, XZ, LW, and CW have filed for a patent from the State Intellectual Property Office of the People's Republic of China, which includes the sequences and the MCDA-LFB method used for detecting *C. albicans* in this manuscript [application number CN 201811580513.9].

## Author Contributions

FaZ, JN, and SH conceived and designed the experiments; FaZ, LY, CW, JW, NG, XZ, and LW performed the experiments; FeZ, JN, LN, NG, XZ, and LW analyzed the data; FaZ, LN, FaZ, and SH contributed reagents, materials, analysis tools; FaZ and LN wrote the manuscript; LN, LY, and SH reviewed the manuscript.

### Conflict of Interest Statement

The authors declare that the research was conducted in the absence of any commercial or financial relationships that could be construed as a potential conflict of interest.

## Supplementary Material

Table S1 Three sets of MCDA primers for *C. albicans* detection.

## Abbreviations

MCDA: Multiple cross displacement amplification
LFB: Lateral flow biosensors
LAMP: Loop-Mediated Isothermal Amplification
LOD: Limit of Detection
ITS II: Internal Transcribed Spacer II
ATCC: American Type Culture Collection
MG: Malachite Green
SGH: Shougang Hospital
FITC: Fluorescein Isothiocyanate
UNG: Uracil-DNA-Glycosylase.
